# Exploring the interaction and driving factors of urban compactness and carbon emission intensity in the Pearl River Delta Urban Agglomeration

**DOI:** 10.1038/s41598-025-94435-x

**Published:** 2025-03-24

**Authors:** Yong Yu, Sameer Kumar, Zhu Ye

**Affiliations:** 1 Asia-Europe Institute, Universiti Malaya, 50603 Kuala Lumpur, Malaysia; 2 Academy of the Zhonghuaminzu Community, Yangtze normal university, Chongqing, China; 3 Faculty of Science, Universiti Malaya, Kuala Lumpur, Malaysia

**Keywords:** Urban compactness, Carbon emission intensity, Coupling coordination, Gray correlation, Pearl River Delta Urban Agglomeration, Climate-change impacts, Sustainability

## Abstract

This paper examines the spatio-temporal interaction and driving factors between urban compactness and carbon emission intensity in the Pearl River Delta Urban Agglomeration from 2010 to 2021. Through the analysis by using comprehensive evaluation, coupling coordination degree model and gray correlation model. Data analysis revealed a steady upward trend in the compactness of the Pearl River Delta Urban Agglomeration. However, there are noticeable regional differences in the compactness of cities. Additionally, carbon emission intensity of urban agglomerations decreases year by year. The rate of change in the carbon emission intensity values varies slightly from city to city. The coupling degree and the coupling coordination degree of urban compactness and carbon emission intensity in the Pearl River Delta Urban Agglomeration are gradually moving towards a coordinated development. Factors such as industrial structure, urbanization level, technological innovation, government intervention and environmental livability, will affect the coupling correlation between urban compactness and carbon emission intensity in the Pearl River Delta Urban Agglomeration. Policy recommendations for city construction should emphasize high-quality urban development and innovative low-carbon urban development models.

## Introduction

Cities are the primary spatial carriers of socio-economic development, with economic densities far exceeding those of other regions. Therefore, the quality of urban development directly affects the comprehensive benefits of regional land use^1^. The rapid pace of urbanization has catalyzed a surge in construction demand. Leading to the expansion of urban areas. This expansion, often characterized by unplanned incremental growth in some cities, can result in negative repercussions known as “sprawl”^2^. Urban sprawl is a phenomenon of inefficient, excessive, and disorderly spatial expansion of urban land that occurs during the rapid urbanization stage^3,4^. It has led to negative effects such as the overconsumption of resources and the destruction of ecological environments^5–8^. In 1973, Dantizg first proposed the concept of the “compact city”,, advocating for reduced travel distances to significantly lower pollution levels and foster sustainable urban growth. Since then, The compact city theory has widely studied and proven to be an effective development model for large cities to alleviate energy scarcity and environmental pollution^9–11^. Meanwhile, the increasing production and consumption activities of humans have generated greenhouse gases such as carbon dioxide, which pose a threat to human survival and development^12^. In 1992, the United Nations released the United Nations Framework Convention on Climate Change, beginning to focus on issues related to climate change^13^. Subsequently, the Kyoto Protocol and the Paris Agreement made unified arrangements for global action on climate change^14^. In 2021, China set forth goals for peaking carbon emissions and achieving carbon neutrality, clarifying the important role of carbon emission reduction in the construction of ecological civilization.

Currently, methods for measuring urban compactness are mainly divided into two categories. One is the single-indicator method, which measures urban compactness through the analysis of urban spatial form^15,16^. This method calculates compactness based on single indicators such as land use^17^, spatial layout^18^, transportation^19^, and social life^20^. This standard for judging urban compactness is overly simplistic, as it only considers the impact of urban form on external compactness and lacks depth^21^. The other category emerged in the early 21st century and places greater emphasis on functional compactness. It constructs a comprehensive evaluation index system for urban compactness^22,23^, considering urban compactness from multiple dimensions including population^24^, land use^25^, economy^9^ and functionality^26^.

Research on carbon emission intensity mainly focuses on the calculation of carbon emissions and the analysis of influencing factors. Methods for calculating carbon emissions include the carbon footprint method, measurement method, remote sensing estimation, building energy consumption simulation, and machine learning^27–31^. In terms of influencing factors, the main factors affecting carbon emission intensity and efficiency include the intensity of environmental regulation^32^, government intervention^33^, industrial structure^34^, urbanization level^35,36^, transportation^37^, and technological level^38^; The impact of different factors on carbon emission intensity and efficiency varies. In addition, many scholars have also studied carbon emission intensity and efficiency from aspects such as income effect^39^, GDP^40^, economic growth^41^, population size^42^, energy intensity^43^, research and development (R&D) intensity^44^, international trade^45^, and industrial^46^.

Urban compactness and carbon emission intensity are closely intertwined, with their interaction influenced by multiple factors, including urban development^47,48^, urban design^49^, transportation infrastructure^50^, and socio-economic behaviors^51–53^. At the same time, factors such as technological innovation^54^, industrial structure^55^, environmental livability^56^, urbanization level^57^, government intervention^58^, and patterns of energy consumption^59^ also determine the contributions of urban density, land use, and economic activities to carbon emission intensity. Overall, although there have been in-depth discussions in the separate fields of urban compactness and carbon emissions, research on the interactive relationship between urban compactness and carbon emission intensity remains relatively limited. Overall, although there have been in-depth discussions in the separate fields of urban compactness and carbon emissions, research on the interactive relationship between urban compactness and carbon emission intensity remains relatively limited.

In conclusion, exploring the interaction between urban compactness and carbon emission intensity is crucial for regional low-carbon sustainable development. The Pearl River Delta Urban Agglomeration is one of the most dynamic economic zones in the Asia-Pacific region and ranks among China’s three major regions with the highest population concentration, strongest innovation capability, and most comprehensive strength. In 2021, the nine cities in the Pearl River Delta had an urban population of 68.7873 million people, accounting for 72.67% of the total urban population in Guangdong Province. The urbanization rate reached as high as 87.5%, and urban development in the region is characterized by high-density agglomeration in core cities and gradual expansion of peripheral cities. Meanwhile, as one of the most economically developed regions, the Pearl River Delta Urban Agglomeration has significant differences in resource and environmental levels among its cities. For example, cities like Shenzhen and Guangzhou have experienced a reduction in green space and an intensification of urban heat island effects, while cities such as Zhuhai and Huizhou emphasize ecological priorities and are committed to low-carbon development. These real-world situations and policy contexts make the Pearl River Delta Urban Agglomeration an important case for study. Therefore, this research analyzes the spatiotemporal evolution characteristics of urban compactness and carbon emission intensity in the Pearl River Delta Urban Agglomeration and investigates the interactive relationship between compactness and carbon emission intensity. It explores the factors influencing their coordinated development level and delves into the synergistic mechanisms between the two. The aim is to fill the existing research gap and promote high-quality regional development.

The key innovations of this study are as follows: (1) By referencing the World Resources Institute’s greenhouse gas inventory guidelines, this study ensures methodological rigor in analyzing carbon emission patterns across the Pearl River Delta, enabling city-specific assessments for more precise insights. (2) Integrating existing research with regional development characteristics, this study establishes a scientifically rigorous, practical, and representative indicator system tailored to the Pearl River Delta’s urban development level. (3) Moving beyond conventional studies that examine only the impact of urban compactness on carbon emissions, this research explores their spatiotemporal interactions and driving mechanisms, offering region-specific insights into sustainable urban development.

The marginal contributions of this study are as follows: (1) Unlike existing studies that primarily examine total changes in urban compactness and carbon emissions, this study adopts per capita, per unit land area, and per unit output efficiency indicators to better capture human-land dynamics. (2) Addressing gaps in the literature, this study provides a deeper analysis of the underlying mechanisms linking urban compactness and carbon emission intensity. (3) While most research emphasizes macro-scale analysis, this study shifts the focus to the prefecture-level city scale within regional urban agglomerations, offering more nuanced insights.

The policy implications of this study are as follows: In the Pearl River Delta region, rapid economic growth and urbanization have made controlling carbon emission intensity essential for sustainable development. This study analyzes how urban compactness affects carbon emission intensity in the region, providing policymakers with insights to optimize resource use and reduce emissions in transportation and buildings while promoting compact urban development. Existing policies focus on urban compactness and green development but lack a systematic understanding of spatiotemporal dynamics. This study addresses this gap by exploring the interactions between urban compactness and carbon emission intensity over time and space. It offers targeted recommendations for urban planning, industrial adjustment, and green initiatives in the region. Additionally, the findings provide valuable references for low-carbon development in other urban agglomerations.

## Research area and data sources

**Fig. 1:**
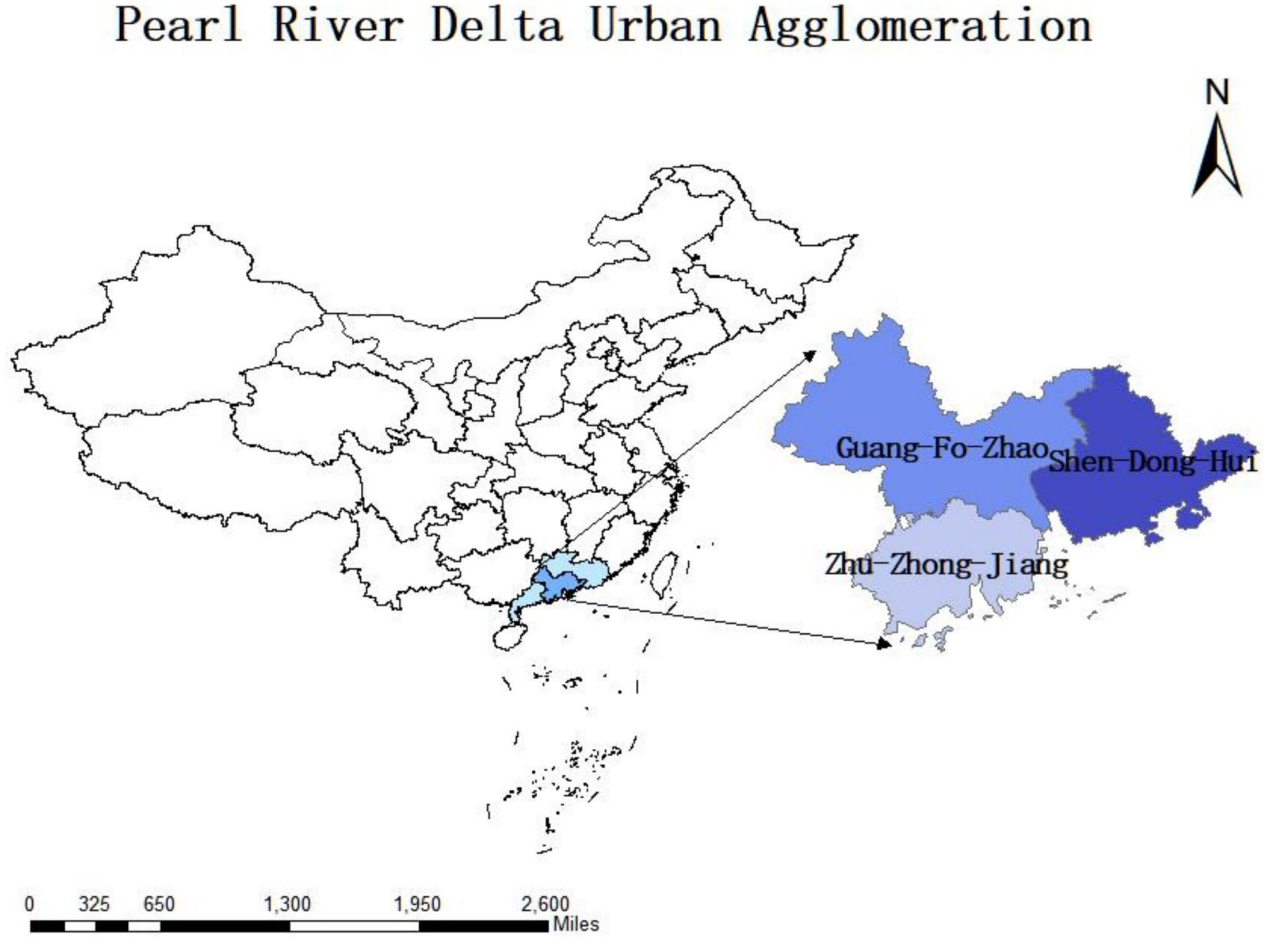
Location of the study area.

The Pearl River Delta Urban Agglomeration (Fig. 1), located in central-southern Guangdong Province, China, borders the South China Sea and spans approximately 55,000 km^2^. It comprises three metropolitan clusters: “Guangzhou-Foshan-Zhaoqing”, “Shenzhen-Dongguan-Huizhou”, and “Zhuhai-Zhongshan-Jiangmen”. As a globally influential hub for advanced manufacturing and modern services, it serves as a key gateway for China’s economic globalization, a national center for scientific and technological innovation, and a critical driver of regional integration. It plays a pivotal role in advancing the economic development of South, Central, and Southwest China. In 2021, the total GDP of the nine cities in the Pearl River Delta Urban Agglomeration exceeded 10 trillion yuan, with Guangzhou, Shenzhen, Foshan, and Dongguan each surpassing 1 trillion yuan. Shenzhen recorded a GDP of 3066.5 billion yuan (6.7% year-on-year growth), ranking third in China, while Guangzhou reached 2823.2 billion yuan (8.1% growth), ranking fourth. The region’s total population reached 78.61 million, an increase of 401,800 from 2020, with Guangzhou, Shenzhen, and Dongguan exceeding 10 million residents each. Foshan, with a population of 9.61 million, experienced the fastest growth in Guangdong, increasing by 93,800 residents. The remaining cities also exhibited varying degrees of population expansion.

This study analyzes the spatiotemporal interaction between urban compactness and carbon emission intensity using population, economic, land use, transportation, and energy consumption data for Pearl River Delta cities from 2010 to 2021. Data sources include the Guangdong Statistical Yearbook, China Urban Construction Statistical Yearbook, China Urban Statistical Yearbook, China Energy Statistical Yearbook, and statistical yearbooks of the nine cities (2011–2022). The missing data was supplemented by trend extrapolation, as detailed in reference^60^. All price related variables are adjusted to 2010 constant prices using the following formula: $\:{X}_{real}=\frac{{X}_{current}}{{P}_{t}}\times\:{P}_{2010}$^61^.

## Research method

### Urban compactness measurement

Breheny defined a compact city as one that curbs uncontrolled urban expansion while enhancing urban center vitality, characterized by high density, efficiency, mixed land use, and well-developed public transportation. Urban compactness is typically measured in terms of physical and functional compactness, with the latter more effectively capturing urban sustainability and livability^62,63^. Based on relevant literature^11,64,65^, this study develops a four-dimensional urban compactness evaluation system—population, economy, land, and transportation—for the Pearl River Delta Urban Agglomeration. A comprehensive weighting approach is employed, integrating both subjective and objective methods to enhance measurement accuracy. The Analytic Hierarchy Process (AHP) is used as the subjective method, ensuring a systematic assessment of factor interrelationships while minimizing redundancy. The entropy method, as the objective approach, assigns higher weights to indicators with greater information content, thereby improving the robustness and representativeness of the urban compactness evaluation^66^.

The Analytic Hierarchy Process (AHP) structures complex problems into a hierarchical framework of interrelated factors, allowing experts to provide quantitative assessments through pairwise comparisons. AHP calculates relative importance weights and performs a consistency test, making it effective for multi-level, multi-dimensional evaluations. The calculation formula can be found in the reference^67^. In contrast, the entropy method derives weights directly from data variation, assigning higher weights to indicators with greater variability and information content. A lower information entropy (e) signifies higher differentiation, indicating a more influential indicator in the evaluation^68^. The calculation formula can be found in the reference^69^. By integrating both subjective expert judgment (AHP) and objective data-driven weighting (entropy method), this study ensures a more balanced and robust urban compactness assessment. By integrating the subjective weights$\:{W}_{1}$ calculated using AHP and the objective weights $\:{W}_{2}$, calculated using the entropy method, the combined weight *Wj* is obtained. The calculation formula is as follows:

1 $$\:{W}_{j}={k}_{1}{W}_{1}+{k}_{2}{W}_{2}$$

where $\:{W}_{1\:}$ and$\:{\:W}_{2}$ represent the indicator weights ascertained through subjective and objective methods. $\:{k}_{1}\:$and $\:{k}_{2}$ denote the contribution weights attributed to these methods. To enhance the precision and reliability of the indicator weights, a combination of subjective and objective methods is used to scientifically allocate weights to the indicator system. In this study, we take $\:{k}_{1}$ = $\:{k}_{2}$ = 0.5^70^.

Based on the weights of the indicators (Table 1), the comprehensive score for urban compactness is derived through linear weighted summation:

2 $$\:{S}_{i}={\sum\:}_{j=1}^{n}{W}_{j}{X}_{ij}$$

where $\:i$ = 1,2,3,…,m; $\:j$ = 1,2,3,…,n; where $\:{W}_{j}$ represents the weight of the j evaluation indicator, and $\:{X}_{ij}$ denotes the standardized value of the i object being evaluated under the j evaluation indicator.

**Table 1:** Index system of urban compactness.

Primary indicators	Secondary indicators	Indicator description	AHP weight	Entropy weight	Comprehensive weight
Population compactness	Urban population density	Urban population/urban area	0.091	0.169	0.130
	Employment population density	Total employed persons/urban area	0.059	0.085	0.072
	Residential population density	Urban population/residential land area	0.035	0.059	0.047
Economic compactness	Output rate per unit area	Total GDP/urban area	0.189	0.102	0.146
	Economic population density	Total GDP/urban population	0.107	0.026	0.066
	Land input intensity	Fixed asset investment/urban area	0.138	0.067	0.102
Land compactness	Urban development and utilization intensity	Built-up area/urban area	0.170	0.113	0.142
	Proportion of urban construction land	Urban construction land area/urban area	0.078	0.086	0.082
	Per capita construction land	Urban construction land area/urban population	0.033	0.089	0.061
Traffic compactness	Road use efficiency	Urban road length/urban road area	0.036	0.053	0.044
	Per capita road traffic facility area	Urban road area/urban population	0.037	0.105	0.071
	Number of taxis per 10,000 people	Number of taxis at year-end/urban population	0.029	0.048	0.030

### Carbon emission intensity measurement

Urban carbon emission intensity is defined as the number of CO_2_ emissions generated per unit of economic output within an urban region. This metric is pivotal for evaluating the quality of a nation’s economic growth and its environmental impact. This study leverages a methodology pioneered by the World Resources Institute, as outlined in their guidelines for creating corporate greenhouse gas emissions inventories. These guidelines recommend segregating emission sources to prevent overlap in counting. The framework for carbon emission calculation encompasses three principal categories^71^, aggregating the emissions from each to derive the total urban carbon output^72^.

Carbon emission intensity, which is the emissions per unit of GDP, stands as a vital index for discerning the relationship between economic expansion and environmental degradation^73^. The formula for calculating carbon emission intensity is delineated as follows:

3 $$\:\text{C}\text{E}\text{I}=\frac{\text{C}\text{B}}{\text{G}\text{D}\text{P}}$$

where $\:\text{C}\text{E}\text{I}$ represents the carbon emission intensity;$\:\:\text{C}\text{B}$ represents the total carbon emissions; and $\:\text{G}\text{D}\text{P}$ represents the Gross Domestic Product.

## Model construction

### Coupling coordination degree model

Coupling, originally a physics concept, describes the interaction and mutual influence between two or more systems. The coupling degree reflects the interdependence and constraints among systems^74–76^ but does not account for their individual development levels^77^. To address this limitation, this study employs the coupling coordination degree model to assess the coordinated development between urban compactness and carbon emission intensity in the Pearl River Delta Urban Agglomeration. This model provides a more comprehensive evaluation of the synergistic relationship between the two subsystems in real-world development^78,79^. The formula is as follows:

4 $$\:\text{C}=2\times\:\sqrt{\frac{\text{U}\times\:\text{R}}{{(\text{U}+\text{R})}^{2}}}$$

5 $$\:\text{T}={\upalpha\:}\text{U}+{\upbeta\:}\text{R}$$

6 $$\:\text{D}=2\times\:\sqrt{\text{C}\times\:\text{T}}$$

where $\:\text{C}$ represents the coupling degree, reflecting the mutual dependence of two systems; $\:\text{U}$ represents the urban compactness within the Pearl River Delta Urban Agglomeration; $\:\text{R}$ represents the carbon emission intensity of the same region; and $\:\text{T}$ represents the comprehensive development index. Considering the equal importance of urban compactness and carbon emission intensity, and given that α + β = 1, α and β are both set to 0.5. $\:\text{D}\:$represents the coupling coordination degree, assessing the synergy level between the two systems.

A higher carbon emission intensity suggests a lower level of low-carbon development, necessitating the application of inverse indicator standardization to carbon emission intensity. According to the literature^80–82^, the coupling coordination degree is divided into an imbalance decline interval and a coordinated development interval. The criteria for division are shown in Table 2.

**Table 2:** Classifications standards of coupling coordination degree.

Type	Coupling coordination stage	Coupling coordination
Coordinated development	High-quality coordination	(0.8 ~ 1.0]
	Intermediate coordination	(0.7 ~ 0.8]
	Primary coordination	(0.6 ~ 0.7]
	Barely coordinate	(0.5 ~ 0.6]
Imbalance decline	Near imbalance	(0.4 ~ 0.5]
	Moderate imbalance	(0.2 ~ 0.4]
	Extreme imbalance	(0 ~ 0.2]

### Grey relational analysis model

The Grey Relational Analysis (GRA) model quantitatively measures the correlation between factors based on the similarity of their development trends. By assessing the shape similarity of data sequences, GRA determines the closeness of relationships more accurately than correlation or regression analysis, making it a more effective method for identifying key influencing factors and their variations within a system^83^.

Common Grey Relational Analysis methods include slope relational degree, Deng’s relational degree, Type-B, and Type-T relational degrees. This study adopts Deng’s relational degree model as it effectively captures relative trends and similarity between variables, while being sensitive to data fluctuations, allowing for a more precise assessment of their relationships. The model is applied to analyze the driving factors and relational degree of variations in the coupling coordination index between urban compactness and carbon emission intensity in the Pearl River Delta Urban Agglomeration. To ensure consistency, data are standardized using the range transformation method, following established literature^84^.

## Measurement results

### Evolution characteristics of urban compactness

**Fig. 2:**
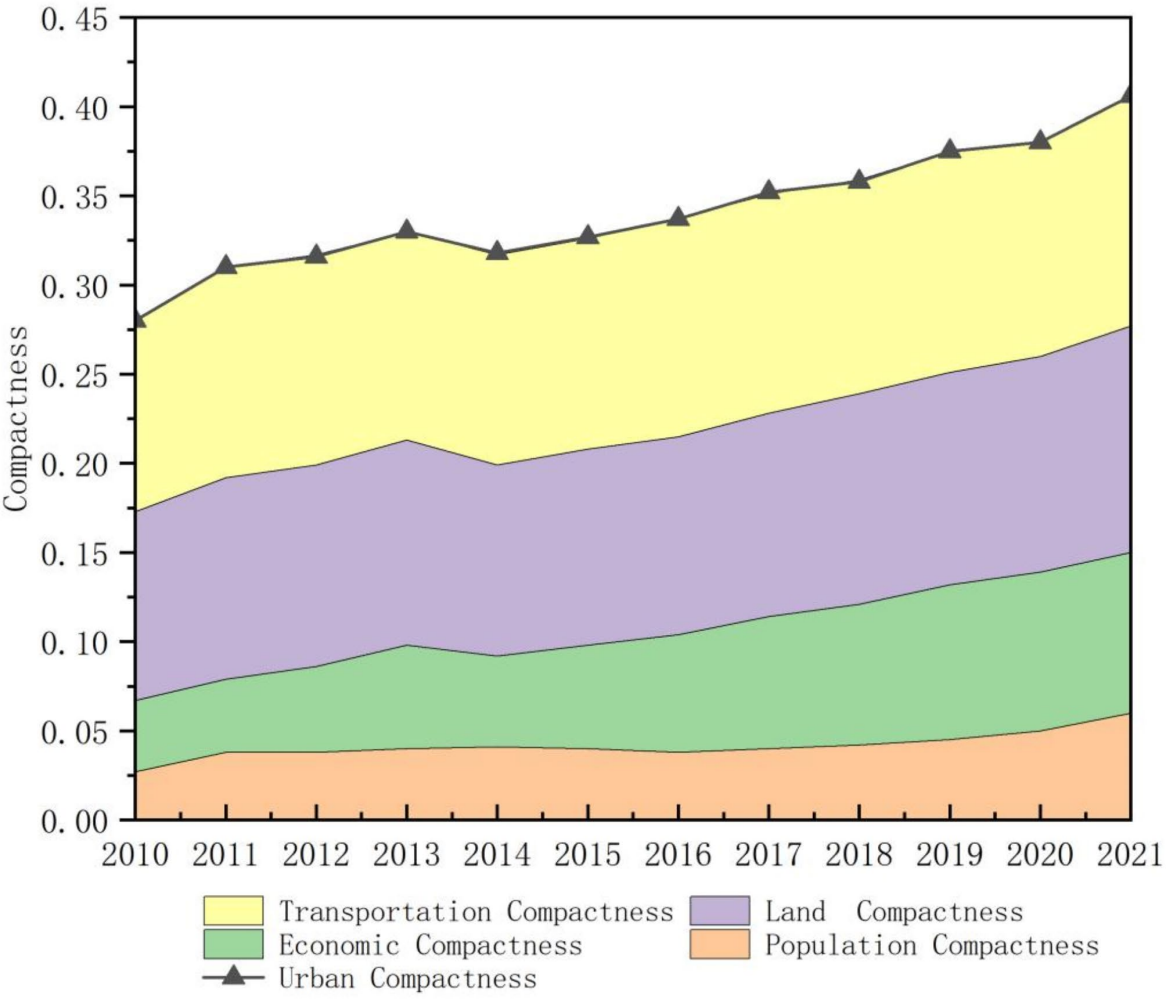
Temporal evolution of urban compactness dimensions in the Pearl River Delta Urban Agglomeration.

From 2010 to 2021, the urban compactness of the Pearl River Delta Urban Agglomeration exhibited a steady upward trend, increasing from 0.280 to 0.406, a 45% overall growth (Fig. 2). This rise was driven by the expansion of urban construction and economic activities.

Among the four dimensions of urban compactness at the end of the study period, land use compactness (31.28%) and transportation compactness (31.77%) contributed the most, followed by economic compactness (22.17%) and population compactness (14.77%). Population compactness remained relatively stable, reflecting constraints imposed by population policies, housing pressure, and spatial carrying capacity, despite the region’s favorable geographical and economic conditions. Economic compactness showed a marked increase, driven by the agglomeration of industries, particularly in light manufacturing, services, and high-tech sectors, which intensified economic activity density. Land compactness also exhibited a steady rise, influenced by recent land use policies. The most significant growth was observed in transportation compactness, which emerged as the dominant driver of urban compactness, highlighting the critical role of transportation infrastructure in regional economic and social development.

**Fig. 3:**
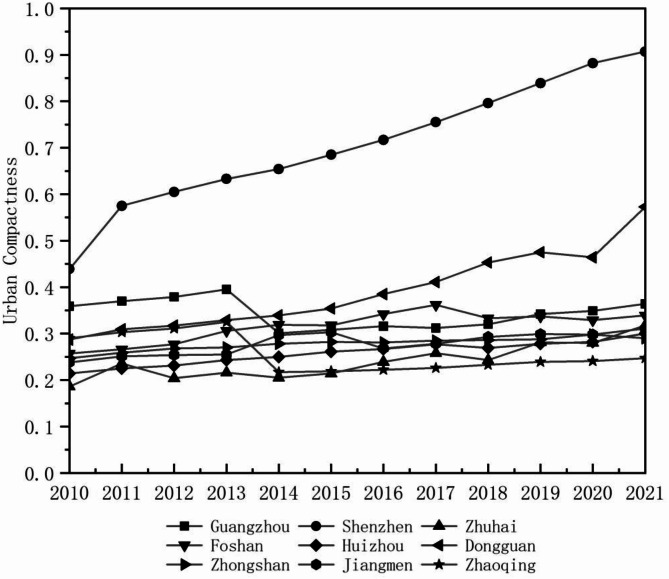
Temporal evolution of urban compactness of different cities in the Pearl River Delta Urban Agglomeration.

The analysis of urban compactness in the Pearl River Delta Urban Agglomeration reveals that Shenzhen and Dongguan experienced the most significant increases. Shenzhen, driven by its strong economic foundation and strategic location, saw a 51.60% growth in compactness from 2010 to 2021, reflecting its long-term focus on urban development since the reform and opening-up. Dongguan, with a 49.91% increase, benefited from its well-developed township economy and the advancement of urbanization and central city primacy. Guangzhou and Zhaoqing also exhibited an upward trend, albeit with fluctuations. Guangzhou experienced a temporary decline in 2013 due to district-level restructuring, but policy adjustments and economic strength facilitated recovery. Zhaoqing, initially demonstrating strong growth, later prioritized economic expansion over resource efficiency and transport infrastructure, leading to a slowdown in compactness improvement. Foshan, Huizhou, Zhongshan, and Jiangmen maintained stable but moderate growth, indicating conscious efforts in urban development, though further land-use intensification is needed. Zhuhai, which had the lowest urban compactness in 2010, showed an overall increasing trend but with significant fluctuations, influenced by limited land area, population size, economic scale, and evolving policy adjustments, despite benefiting from special economic zone status (Fig. 3).

**Fig. 4:**
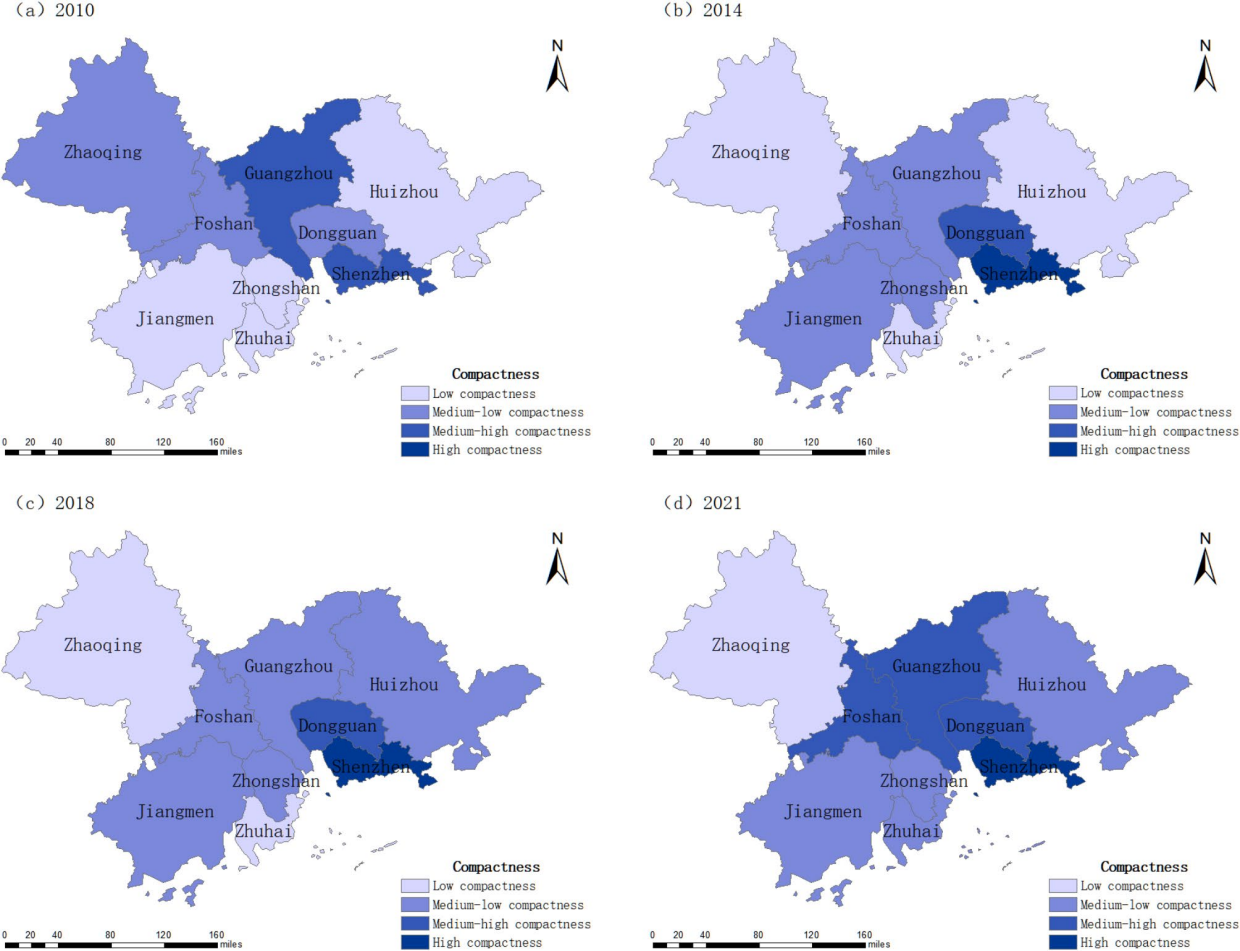
Spatial evolution of compactness of Pearl River Delta Urban Agglomeration.

After analyzing the cities within the Pearl River Delta Urban Agglomeration across the spatial dimension, compactness data from four time points—2010, 2014, 2018, and 2021—were selected. Based on the compactness scores, the nine cities in the Pearl River Delta were classified into four levels: low compactness (0.186–0.254), medium-low compactness (0.254–0.338), medium-high compactness (0.338–0.575), and high compactness (0.575–0.910). The spatial evolution characteristics of urban compactness within the urban agglomeration were then visualized using ArcGIS 10.6 (Fig. 4).

Spatial analysis reveals a “high in the center, low in the periphery” pattern. Central cities, serving as the core of the Pearl River Delta, exhibit higher compactness due to industrial concentration, employment opportunities, public services, and efficient transportation networks. Urban renewal initiatives, industrial park optimization, and mixed-use development further enhance compactness. In contrast, peripheral cities face constraints such as weaker economic foundations, lower land development intensity, dispersed urban expansion, and inefficient land use, resulting in lower compactness.

In 2010, most cities in the Pearl River Delta Urban Agglomeration were in a medium-low compactness state, while Guangzhou and Shenzhen were in a medium-high compactness state. This indicates that most cities were in the early stages of construction with weak population agglomeration effects. Guangzhou and Shenzhen had higher compactness than other cities due to their better economic and urban construction levels. In 2014, the differentiation of urban compactness intensified. Shenzhen had high compactness due to rapid urbanization and a regional agglomeration effect. Dongguan had medium-high compactness due to its advantageous geographical location attracting population inflows. Zhongshan, Foshan, and Jiangmen had medium-low compactness due to stable economic development and industrial layout. Guangzhou’s compactness decreased as urban construction expanded faster than population and economic growth. Zhuhai, Zhaoqing, and Huizhou maintained the same level of compactness. In 2018, Huizhou improved its compactness to a medium-low level by attracting population and investment through its special industries and geographical advantages. Other cities maintained their compactness levels. In 2021, due to rapid development and improved infrastructure. Zhuhai significantly increased its compactness to a medium-low level due to geographical advantages and policy adjustments. Other cities maintained their compactness levels.

### Evolution characteristics of carbon emission intensity

The calculation of carbon emission intensity in the Pearl River Delta Urban Agglomeration shows that the overall carbon emission intensity of the region has been declining (Table 3). The average value decreased from 1.569 tons per 10,000 yuan in 2010 to 0.825 tons per 10,000 yuan in 2021, a reduction of 47.42%. This significant decline in carbon intensity is mainly attributed to the optimization of industrial structure and the improvement of energy utilization efficiency following the implementation of relevant policies. As a result, the Pearl River Delta Urban Agglomeration has placed greater emphasis on environmental protection while developing its economy. The variance of carbon emission intensity decreased from 1.095 in 2010 to 0.304 in 2021, a reduction of 72.24%. This is due to the improved economic development levels across cities and the deepening of integrated development concepts. The differences in carbon emission intensity among regions in the Pearl River Delta Urban Agglomeration have gradually narrowed.

**Table 3:** Overall change trend of carbon emission intensity in Pearl river Delta urban agglomeration, 2010–2021 (Carbon emission intensity unit: t CO_2_/10,000 CNY).

	2010	2011	2012	2013	2014	2015	2016	2017	2018	2019	2020	2021
Mean	1.569	1.418	1.280	1.236	1.163	1.130	1.055	1.004	0.913	0.911	0.905	0.825
Median	1.747	1.731	1.431	1.392	1.165	1.333	1.209	1.126	0.950	1.002	1.029	0.867
Max	3.125	3.045	2.549	2.513	2.483	2.313	2.164	2.252	1.808	1.799	1.743	1.822
Min	0.429	0.386	0.353	0.305	0.305	0.273	0.274	0.232	0.229	0.213	0.219	0.212
Variance	1.095	0.903	0.660	0.666	0.601	0.548	0.468	0.468	0.342	0.348	0.327	0.304

**Fig. 5:**
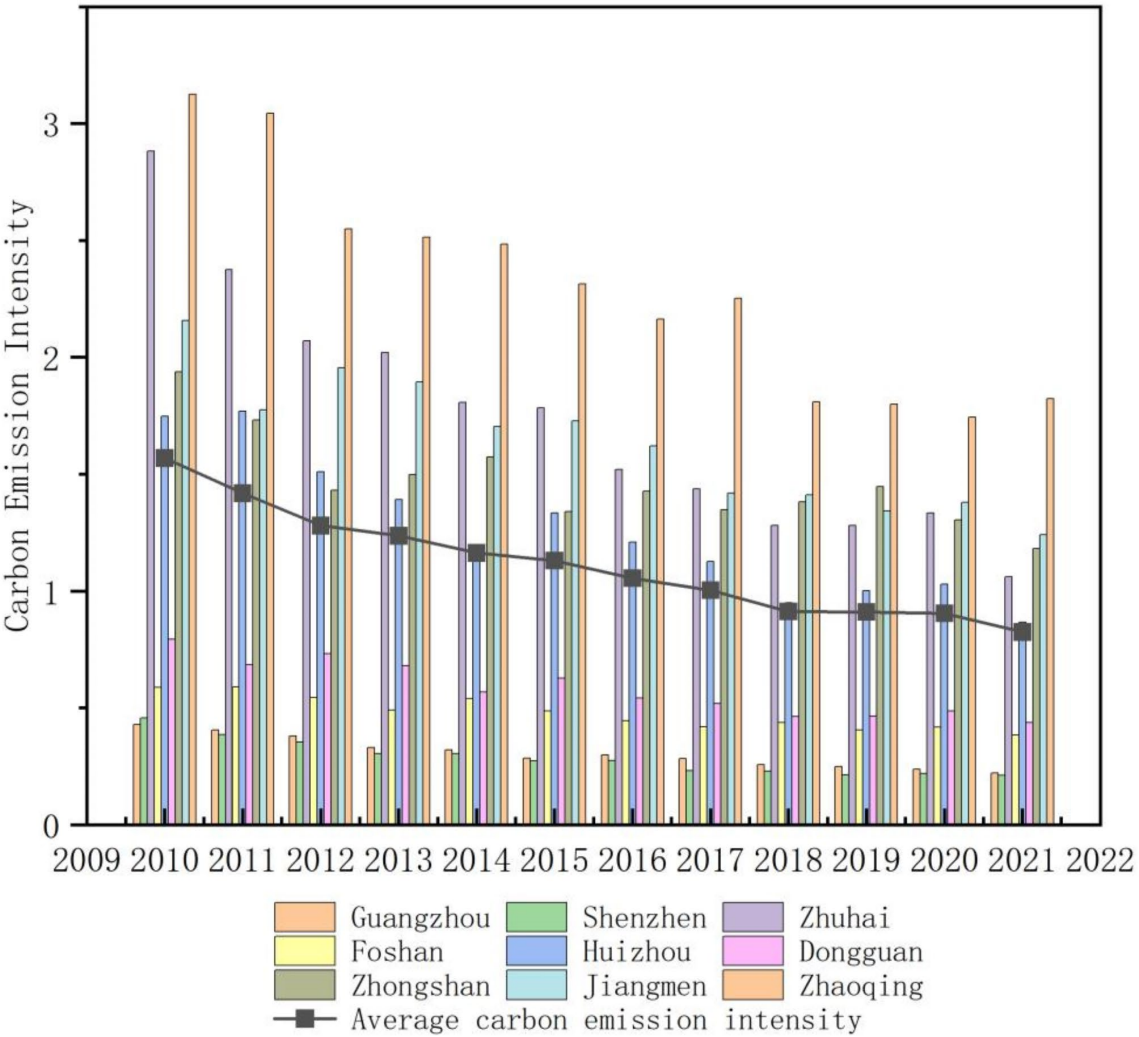
Time evolution of carbon emission intensity in Pearl River Delta Urban Agglomeration.

The annual computation of carbon emission intensity for each city (Fig. 5) reveals a consistent declining trend in overall carbon emission intensity across the Pearl River Delta Urban Agglomeration. Among the cities, Shenzhen shows the lowest Carbon Emission Intensity values, while Zhaoqing has comparatively higher levels. Additionally, fluctuations in carbon emission intensity were observed in the Pearl River Delta Urban Agglomeration in various years. Dongguan witnessed the most significant fluctuation frequency, succeeded by Foshan, Huizhou, and Zhongshan, while Guangzhou and Shenzhen recorded the most stable emission levels, showcasing the lowest frequencies of fluctuation.

Analyzing the comprehensive trends in carbon emission intensity across the Pearl River Delta Urban Agglomeration, the evolution can be categorized into three distinct stages:

1. Phase one: high carbon emission phase (2010–2013).

During this period, the Pearl River Delta Urban Agglomeration experienced rapid industrialization and urbanization, with a manufacturing sector heavily reliant on high-energy consumption. Despite increasing urban compactness, inefficient industrial layouts, inadequate transportation infrastructure, and low energy efficiency limited its potential to mitigate carbon emissions. Instead, the concentration of industrial activities may have exacerbated carbon emission intensity. The region’s overall economic strength remained relatively weak, and energy utilization efficiency was low, resulting in persistently high carbon emissions.

(2) Phase two: continuous emission reduction phase (2014–2017).

During this stage, cities underwent industrial restructuring, gradually transitioning toward service and high-tech industries. The implementation of environmental policies and green technologies facilitated a decline in carbon emissions. Concurrently, urban infrastructure improvements, public transportation development, and optimized land use planning enhanced urban compactness, aligning compact development with emission reduction objectives. Reduced transportation emissions and improved energy efficiency contributed to a gradual decline in carbon emission intensity. With robust economic growth and significant advancements in energy structure and efficiency, carbon emission intensity continued to decrease steadily.

(3) Phase three: low carbon optimization stage (2018–2021).

During this period, cities entered a mature stage of low-carbon development, with widespread application of green technologies and clean energy becoming the primary energy source. Carbon emissions in economic activities were minimized. The relationship between urban compactness and carbon emission intensity became more optimized. Highly compact urban layouts further reduce energy consumption and transportation carbon emissions while maintaining efficient resource utilization, with carbon emission intensity at very low levels or approaching zero. The quality of economic development was high, and the energy supply structure became more scientific and rational, with overall carbon emission intensity values in an optimal range.

By analyzing the carbon emission intensity of each city in the Pearl River Delta Urban Agglomeration, it can be observed that the high-value areas of carbon emission intensity are consistently located in the western part of the urban agglomeration. Cities such as Zhaoqing, Jiangmen, Zhongshan, and Zhuhai are dominated by high-energy-consuming industries like manufacturing, resulting in higher carbon emission intensity. In contrast, Huizhou, Foshan, Guangzhou, and Shenzhen are primarily driven by non-energy-intensive industries such as high-tech and finance, leading to relatively lower carbon emission intensity. The significant reduction in carbon emission intensity in Zhuhai and Zhaoqing in 2021 can be attributed to their increasing focus on environmental protection and sustainable development over the years, which has progressively enhanced their low-carbon development levels.

**Fig. 6:**
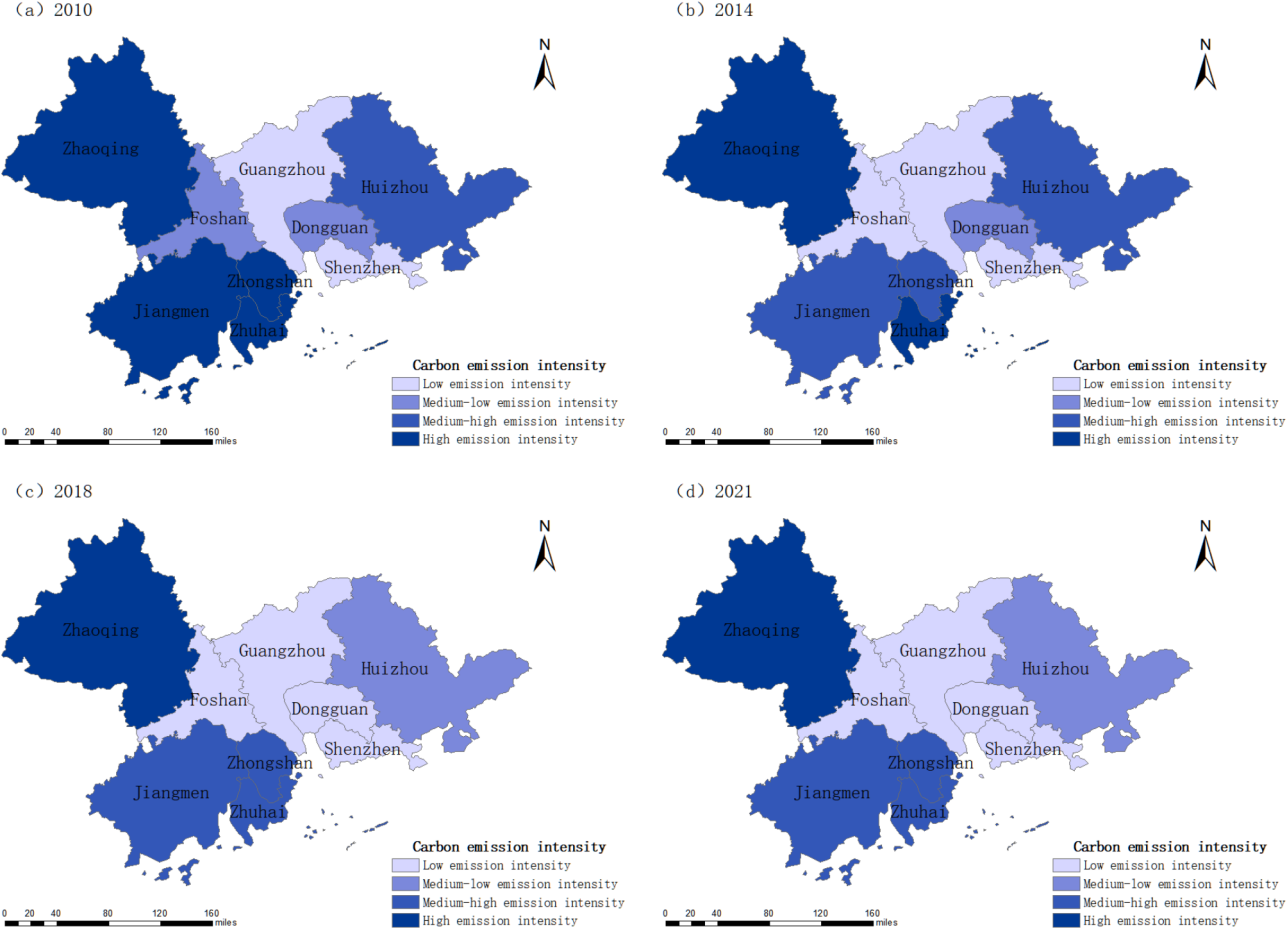
Spatial evolution of carbon emission intensity in Pearl River Delta Urban Agglomeration.

Based on the values of carbon emission intensity, the emissions were categorized into four levels: low emission intensity (0.212–0.544), medium-low emission intensity (0.544–1.029), medium-high emission intensity (1.029–1.747), and high emission intensity (1.747–3.125). The spatial distribution of carbon emission intensity was visualized using ArcGIS 10.6 (Fig. 6).

The spatial distribution of carbon emission intensity in the Pearl River Delta Urban Agglomeration follows a pattern of “higher in peripheral areas and lower in central areas.” Peripheral cities, dominated by traditional manufacturing, exhibit higher carbon emission intensity due to outdated industrial structures, lower technological advancements, and weaker enforcement of carbon reduction policies. In contrast, central cities, characterized by a strong presence of service and high-tech industries, have actively promoted energy-efficient industries and implemented targeted, well-enforced low-carbon policies. As a result, these cities demonstrate more advanced low-carbon development and consistently lower carbon emission intensity.

In 2010, cities such as Zhaoqing, Jiangmen, Zhongshan, Zhuhai, and Huizhou exhibited high or moderately high carbon emission intensities due to their industrial structures and urban expansion. Foshan and Dongguan, with a focus on light industry, had moderate to low emissions, while Guangzhou and Shenzhen, dominated by low-energy-consuming industries, maintained low emission levels. By 2014, Foshan reduced its emissions through environmental reforms and industrial upgrading, while Jiangmen and Zhongshan, with significant progress in industrial upgrading, saw their carbon emission intensity decrease to moderately high levels. In 2018, Dongguan’s shift towards low-energy light industries and clean energy further lowered emissions, while Huizhou and Zhuhai improved through industrial restructuring and environmental regulations. By 2021, carbon emission intensity across the region remained stable at 2018 levels, with no further changes among cities.

### Analysis of the coupling coordination degree

#### Evolution characteristics of the coupling coordination

**Fig. 7:**
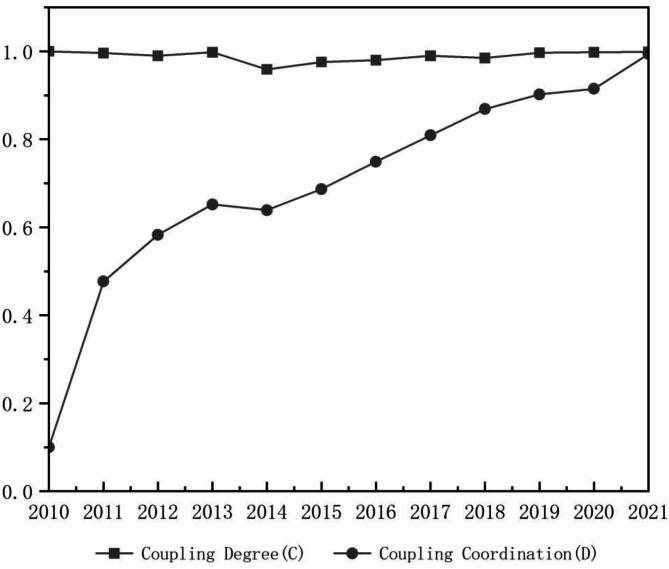
Coupling and coordinated changes of Pearl River Delta Urban Agglomeration compactness and carbon emission intensity, 2010–2021.

Figure 7 illustrates the coupling coordination level between urban compactness and carbon emission intensity within the Pearl River Delta Urban Agglomeration from 2010 to 2021. The coupling coordination degree between urban compactness and carbon emission intensity increased from 0.100 in 2010 to 0.994 in 2021, showing a stable upward trend (Table 4). This progression suggests that the relationship between the two systems has gradually evolved from extreme imbalance to high-quality coordination.

**Table 4:** Coordinated changes in the coupling of compactness and carbon emission intensity in the Pearl river Delta urban agglomeration, 2010–2021.

	Coupling degree (C)	Coupling coordination (D)	Coupling coordination degree
2010	1.000	0.100	Extreme imbalance
2011	0.996	0.477	Near imbalance
2012	0.990	0.583	Barely coordinate
2013	0.998	0.652	Primary coordination
2014	0.959	0.639	Primary coordination
2015	0.976	0.687	Primary coordination
2016	0.980	0.749	Intermediate coordination
2017	0.990	0.809	High-quality coordination
2018	0.985	0.869	High-quality coordination
2019	0.997	0.902	High-quality coordination
2020	0.998	0.915	High-quality coordination
2021	0.999	0.994	High-quality coordination

**Fig. 8:**
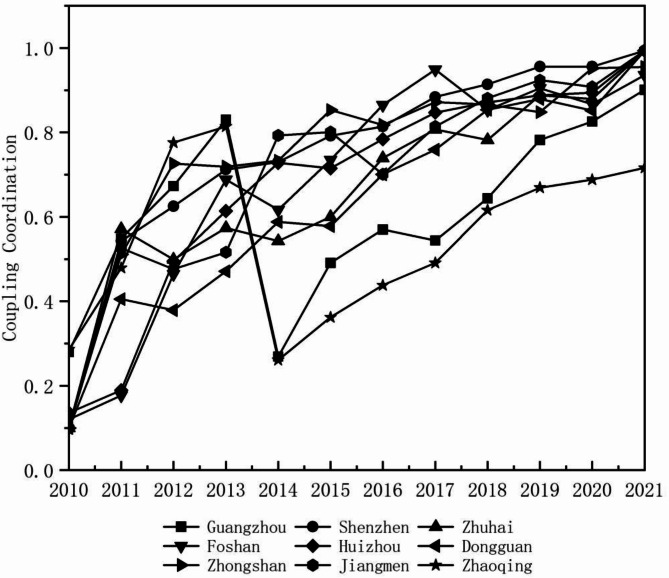
Coupling coordinated changes of urban compactness and carbon emission intensity in the Pearl River Delta Urban Agglomeration, 2010–2021.

Figure 8 illustrates the evolving relationship between urban compactness and carbon emission intensity in the Pearl River Delta Urban Agglomeration, transitioning from imbalance to coordination.

In 2010, Guangzhou and Zhaoqing exhibited the highest coupling coordination, benefiting from rational urban construction and industrial structures. However, Guangzhou’s coordination declined in 2014 due to district restructuring, increased reliance on high-carbon energy, and inefficient industrial energy use. Subsequent industrial and urban adjustments restored high-quality coordination. Zhaoqing, with the largest land area and richest ecological resources in the Greater Bay Area, initially had rational urban construction and industrial structure, resulting in high coordination. Until 2013, Zhaoqing’s coordination weakened as its secondary sector expanded to 50%, intensifying carbon emissions and disrupting spatial planning. The city later improved through its transition to new energy industries, reaching medium coordination by 2021.

Shenzhen, Zhuhai, Foshan, Dongguan, Zhongshan, Huizhou, and Jiangmen showed fluctuating but overall increasing coordination trends, achieving high-quality coordination by 2021. Initially, rapid land expansion and high-carbon industrial growth constrained low-carbon development. However, as these cities optimized energy structures and shifted to emerging industries, carbon emissions per unit of GDP declined, and high-density development enhanced economic efficiency, driving the coordination upward.

**Fig. 9:**
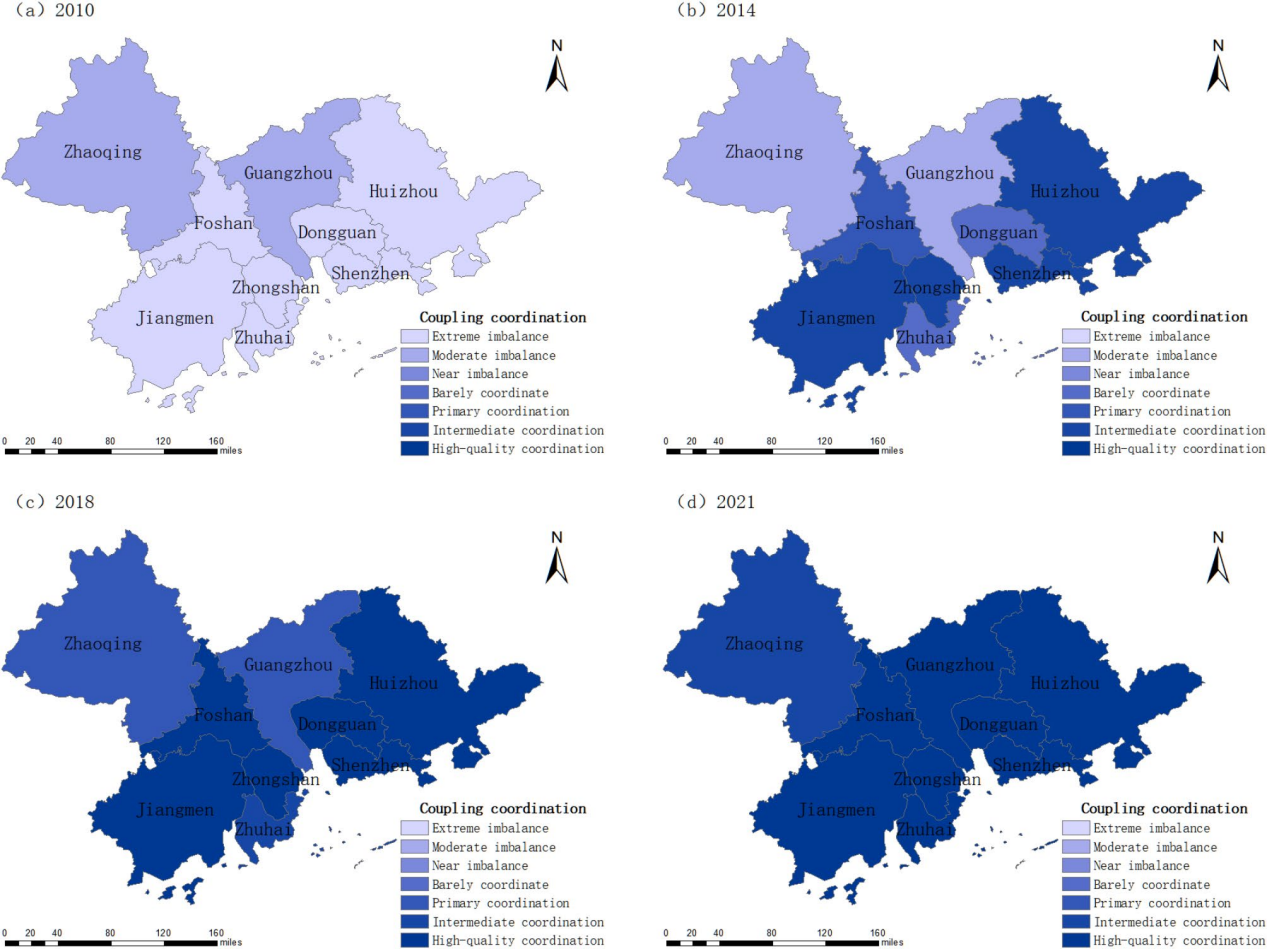
Coordinated changes in the coupling of compactness and carbon emission intensity in the Pearl River Delta Urban Agglomeration, 2010–2021.

The relationship between urban compactness and carbon emission intensity in the Pearl River Delta Urban Agglomeration has progressed from imbalance to coordination (Fig. 9).

In 2010, Guangzhou and Zhaoqing exhibited moderate imbalance, while other cities experienced extreme imbalance. Guangzhou’s industrial upgrading, mature urban planning, and strict environmental policies (e.g., the “Blue Sky Plan”) reduced carbon emissions. Zhaoqing, with a lower share of heavy industry and controlled urban expansion, mitigated high-carbon emissions. In contrast, other cities, lacking coordinated planning, faced significant spatial and carbon emission imbalances. By 2014, five cities achieved coordination, with Huizhou, Shenzhen, Jiangmen, and Zhongshan reaching high-quality coordination due to optimized industrial structures, compact development, and policy-driven emission reductions, while some manufacturing-dependent cities still struggled. In 2018, Guangzhou and Zhaoqing remained at the primary coordination stage due to their industrial structure and suburban low-density development, while Zhuhai advanced to an intermediate coordination stage, and other cities achieved high-quality coordination through industrial transformation and compact urban planning. By 2021, Zhaoqing progressed to intermediate coordination but lagged in achieving high-quality coordination due to reliance on traditional manufacturing, dispersed urban form, and limited low-carbon policy implementation. Other cities successfully advanced in low-carbon development and compact urban growth. Overall, the Pearl River Delta Urban Agglomeration has reached a relatively high level of coupling and coordination.

### Identification of driving factors

The coupling coordination degree between urban compactness and carbon emission intensity in the Pearl River Delta Urban Agglomeration is influenced by multiple factors including social, economic, and policy aspects, yet the spatial differentiation mechanism remains unclear. The Pearl River Delta Urban Agglomeration is characterized by rapid economic development, swift industrial upgrading, high levels of urbanization, strong policy intervention, and significant ecological pressure, all of which directly affect the coordinated development of the two. Therefore, this paper selects the following influencing factors for analysis:

1. Industrial structure. Industrial structure reflects the composition of urban economic activities, with significant differences in carbon emission characteristics among different industries^85^. The Pearl River Delta Urban Agglomeration has transitioned from traditional manufacturing to high-end manufacturing and service industries, reducing the proportion of high-energy-consuming industries and significantly influencing the relationship between urban carbon emission intensity and compactness. This paper uses the proportion of the tertiary sector to measure industrial structure^82^.
2. Urbanization level. The impact of urbanization on carbon emission intensity varies with development stages, and population agglomeration promotes compact urban development^86^. The Pearl River Delta Urban Agglomeration is highly urbanized with well-developed infrastructure and public transportation. Through efficient land use and transportation planning, traffic-related carbon emissions have been reduced, and urban compactness and resource efficiency have been enhanced. This paper uses the proportion of urban population to measure the level of urbanization^87^.
3. Technological innovation. Technological innovation directly reduces carbon emission intensity by improving industrial technology and drives high-quality urban development^88^. The Pearl River Delta Urban Agglomeration has developed low-carbon technologies such as new energy and intelligent transportation, enhancing resource utilization efficiency and the coordination of low-carbon urban development. This paper uses the proportion of R&D expenditure in GDP to measure technological innovation^89^.
4. Government intervention. Reasonable government intervention facilitates the rational allocation of resources and promotes the coordinated development of urban compactness and carbon emission intensity^90^. Local governments in the Pearl River Delta Urban Agglomeration have driven low-carbon transitions through policy regulation, carbon trading mechanisms, and green building standards. This paper uses general public budget expenditure to represent government intervention^91^.
5. Environmental livability. Urban parks effectively reduce carbon emissions, and superior ecological environments attract population and industrial agglomeration, serving as an important reference for the level of compact urban construction^92^. The Pearl River Delta Urban Agglomeration has promoted compact development and carbon emission reduction through low-carbon community construction, improved air quality, and increased green space. This paper uses per capita park area to reflect the environmental level^93^.

**Table 5:** Correlation of drivers of the degree of development of compactness and carbon emission intensity decoupling in Pearl river Delta cities.

	Industrial structure	Urbanization level	Technological innovation	Government intervention	Environmental livability
Guangzhou	0.597	0.584	0.656	0.657	0.594
Shenzhen	0.761	0.709	0.759	0.746	0.689
Zhuhai	0.716	0.653	0.788	0.812	0.699
Foshan	0.662	0.608	0.648	0.571	0.723
Huizhou	0.645	0.621	0.699	0.765	0.668
Dongguan	0.586	0.618	0.837	0.763	0.689
Zhongshan	0.788	0.733	0.739	0.840	0.761
Jiangmen	0.688	0.645	0.796	0.827	0.761
Zhaoqing	0.696	0.643	0.650	0.639	0.589

**Table 6:** Correlation of drivers of the degree of development of compactness and carbon emission intensity decoupling in Pearl river Delta cities.

	Correlation degree	Rank
Industrial structure	0.795	2
Urbanization level	0.788	4
Technological innovation	0.796	1
Government intervention	0.671	5
Environmental livability	0.791	3

Using the Grey Relational Analysis Model, this study quantifies the correlation between the coupling coordination degree and its driving factors (Table 5). A higher correlation degree indicates a greater influence on the coupling coordination degree. The results reveal that industrial structure, urbanization level, technological innovation, government intervention, and environmental livability all have correlation degrees exceeding 0.5, signifying a robust relationship. From an aggregate perspective, the order of correlation strength is as follows: technological innovation > industrial structure > environmental livability > urbanization level > government intervention (Table 6).

1. Industrial structure. Shenzhen, Zhuhai, and Zhongshan have association scores above 0.7, with Zhongshan reaching the highest score of 0.788, indicating that the proportion of the tertiary sector has the strongest association with urban compactness and carbon emission reduction. Foshan, Huizhou, Jiangmen, and Zhaoqing have association scores between 0.6 and 0.7, showing a strong correlation. Guangzhou and Dongguan have scores between 0.5 and 0.6, with Dongguan having the lowest score of 0.586, indicating the least sensitivity to the proportion of the tertiary sector. This is related to the slower industrial transformation and higher proportion of energy-intensive industries in Guangzhou and Dongguan.
2. Urbanization level. Shenzhen and Zhongshan have association scores above 0.7, with Zhongshan having the highest level of urbanization and being most sensitive to the coupling coordination. Zhuhai, Foshan, Huizhou, Dongguan, Jiangmen, and Zhaoqing have association scores between 0.6 and 0.7, showing a strong correlation. Guangzhou has the lowest association score of 0.584, indicating the least sensitivity to urbanization. This is related to Guangzhou’s urban expansion model and uneven resource allocation as a megacity, which limits the regulatory effect of urbanization on carbon emissions.
3. Technological innovation. Dongguan has the highest association score for technological innovation at 0.837, indicating the most significant promotion of coupling coordination. Shenzhen, Zhuhai, Zhongshan, and Jiangmen have association scores between 0.7 and 0.8, showing a strong correlation. Guangzhou, Foshan, Huizhou, and Zhaoqing have scores between 0.6 and 0.7, indicating that technological innovation has a less noticeable effect on these cities. This is related to insufficient technological innovation investment or low technology conversion efficiency in these cities.
4. Government intervention. Zhuhai, Zhongshan, and Jiangmen have association scores above 0.8, while Shenzhen, Huizhou, and Dongguan are between 0.7 and 0.8. Guangzhou and Zhaoqing have scores between 0.6 and 0.7, and Foshan has the lowest score of 0.571, indicating the least sensitivity to coupling coordination. This is related to Foshan’s relatively insufficient policy implementation and resource allocation efficiency.
5. Environmental livability. Zhongshan, Jiangmen, and Foshan have association scores above 0.7, indicating a strong correlation between environmental livability and coupling coordination. Shenzhen, Zhuhai, Huizhou, and Dongguan have scores between 0.6 and 0.7, while Guangzhou and Zhaoqing are between 0.5 and 0.6, with Zhaoqing having the lowest score of 0.589. This is related to Zhaoqing’s good ecological foundation but underutilization of its role in coupling coordination. Zhongshan and Jiangmen, with close geographical and cooperative ties, show a much stronger promotion of coupling coordination by environmental livability than other cities.

## Conclusions

The relationship between urban compactness and carbon emission intensity in the Pearl River Delta is shaped by various factors, including technological innovation, industrial structure, environmental livability, urbanization level, and government intervention. Increased urban compactness, when supported by appropriate regulations and innovations, has the potential to reduce carbon emission intensity, thereby achieving a balance between efficient resource utilization and low-carbon development. However, without effective policies and technological innovations, higher urban compactness can exacerbate energy consumption and environmental pollution. Based on the analysis of urban compactness, carbon emission intensity, and the driving factors within the Pearl River Delta Urban Agglomeration, the following conclusions are drawn:

1. Urban compactness trends. The overall trend in urban compactness across the Pearl River Delta is increasing, with Shenzhen exhibiting consistent and robust growth. In contrast, Guangzhou, Dongguan, and Zhaoqing display a cyclical pattern of rising, then falling, followed by another increase. Meanwhile, Zhuhai, Foshan, Huizhou, Zhongshan, and Jiangmen show a fluctuating upward trend.
2. Carbon emission intensity. The carbon emission intensity across the Pearl River Delta demonstrates a general yearly reduction trend. The average carbon emission intensity decreased from 15,690 tons per billion yuan in 2010 to 8,250 tons per billion yuan in 2021, indicating substantial progress towards a low-carbon economy. This trend can be categorized into three stages: the high carbon emission stage (2010–2013), the continuous reduction stage (2014–2017), and the low-carbon optimization stage (2018–2021).
3. Coupling coordination. The urban compactness and carbon emission intensity in the Pearl River Delta have been mostly coordinated throughout most years, with only a few years showing imbalance. Specifically, the coupling coordination degree between urban compactness and carbon emission intensity in Guangzhou and Zhaoqing shows an initial increase, followed by a decrease, and then another rise. Other cities exhibit a fluctuating upward trend.
4. Driving factors. The analysis of driving factors shows that industrial structure, level of urbanization, technological innovation, government participation, and environmental livability have strong correlations with changes in the coupling coordination degree. Based on the overall data of the urban agglomeration, the influence of driving factors from strongest to weakest is as follows: technological innovation < industrial structure < environmental livability < level of urbanization < government participation. Additionally, Zhongshan’s UC and carbon emission intensity coupling coordination degree is highly associated with changes in driving factors. Guangzhou, Zhuhai, Huizhou, and Jiangmen are more sensitive to changes in government participation. Shenzhen and Zhaoqing are more sensitive to changes in industrial structure. Foshan more is sensitive to changes in environmental livability.

### Policy implications

Based on the above findings, this study suggests that the Pearl River Delta Urban Agglomeration could adopt the following measures to foster high-quality urban and economic development:

1. Promote coordinated development of urban functional compactness. The Pearl River Delta Urban Agglomeration faces high population density and significant pressure on resources and the environment due to rapid urbanization. This agglomeration has exacerbated the pressure on urban resources and the environment. Insufficient public transportation networks and intercity transport connections have led to increased dependence on high-carbon travel and higher carbon emissions from commuting between cities. The government should promote integration of production and urban functions, optimize public transport layouts, and improve land use efficiency. Guangzhou and Shenzhen need to coordinate the development of central urban areas with peripheral functional zones to reduce spatial agglomeration. Dongguan and Foshan should optimize transport networks to improve travel efficiency. Huizhou, Jiangmen, and Zhaoqing need to enhance land planning to prevent uncontrolled urban sprawl.
2. Optimize industrial structure to promote high-quality economic development. Although the Pearl River Delta Urban Agglomeration has advantages in the service and high-tech industries, manufacturing remains the backbone of the economy. In particular, manufacturing upgrades in Zhaoqing, Jiangmen, and other places lag behind, and small and medium-sized enterprises face difficulties in green transformation, which hinders the development of a low-carbon economy. The government should strengthen energy-saving and emission-reduction supervision, promote green manufacturing and the application of clean energy, and build a low-carbon technology innovation system. Guangzhou and Shenzhen need to increase R&D support to promote green transformation of enterprises. Dongguan, Foshan, and Zhongshan should provide technical upgrade subsidies to transform high-pollution enterprises and reduce carbon emission intensity.
3. Strengthen local government governance capacity to promote integrated development. Although the Pearl River Delta Urban Agglomeration has low-carbon development policies, it still faces challenges in regional coordination and policy implementation. Cities operate independently, and the coordinated governance mechanism is not sound, leading to inefficient resource allocation. The government should improve low-carbon development systems, promote low-carbon awareness, strengthen regional coordinated governance, enhance policy implementation, and create a green social atmosphere. Shenzhen, Guangzhou, and other economically developed cities need to strengthen policy linkages to improve resource allocation efficiency. Huizhou, Jiangmen, and Zhaoqing need to enhance service capabilities, strengthen regional cooperation, and promote integrated development.
4. Construct green barriers to increase the carbon sink ratio of the urban agglomeration. Rapid urbanization has led to a reduction in green space and damage to ecosystems in the Pearl River Delta, resulting in insufficient carbon sink capacity. Guangzhou and Shenzhen have seen a decrease in green land. Foshan, Dongguan, and Zhaoqing have heavy industrial pollution, limiting ecological livability. The government should develop an ecological plan for the urban agglomeration, coordinate the construction of ecological space, and build a green ecological barrier. Shenzhen and Guangzhou should expand green space and open areas. Foshan, Dongguan, and Zhaoqing need to add green belts to reduce pollution pressure. Zhuhai, Huizhou, Jiangmen, and Zhongshan should prioritize ecological protection to promote green development and improve environmental livability.

### Research limitations

Despite being the first to assess the spatiotemporal interactions between urban compactness and carbon emission intensity and their driving factors in the Pearl River Delta Urban Agglomeration, this study has several limitations. First, although this study selected a comprehensive set of indicators, some factors still need to be evaluated due to the different development stages and influencing factors of cities. Second, our research is based on the Pearl River Delta Urban Agglomeration, but given the varying levels of development among urban agglomerations in China, it is difficult to apply the conclusions and policy recommendations to all urban agglomerations.

## Data Availability

The data used in this study are available upon request from the corresponding author.
